# Giant Right Coronary Artery Aneurysm: A Case Report

**DOI:** 10.7759/cureus.3609

**Published:** 2018-11-19

**Authors:** Iqra Saghir, Zunera Moeen, Ghanwa Saghir, Shida Bangash, Sara Tariq, Shamima Akter

**Affiliations:** 1 Internal Medicine, King Edward Medical University / Mayo Hospital, Lahore, PAK; 2 Obstetrics and Gynecology, Pakistan Institute of Medical Sciences, Islamabad, PAK; 3 Internal Medicine, Jinnah Hospital Lahore (JHL)/Allama Iqbal Medical College (AIMC), Lahore, PAK; 4 Internal Medicine, Sher-E-Bangla Medical College, Barisal, BGD

**Keywords:** coronary artery, coronary angiography, aneurysms, kawasaki disease, cardiac catheterization, right coronary artery

## Abstract

Coronary artery aneurysms are not very uncommon but 'giant' coronary artery aneurysms are rare, with a reported prevalence of 0.02% to 0.2%. Coronary artery aneurysm may be symptomatic or asymptomatic depending on their size and location but it is very unusual for a giant coronary artery aneurysm to be asymptomatic. Here, we present a case in which the giant coronary artery aneurysm remained undiagnosed and asymptomatic for several years.

## Introduction

Coronary artery aneurysms may be symptomatic or asymptomatic depending upon the size and location of the aneurysm. It is very unusual for an untreated giant arterial aneurysm to be asymptomatic for a very long time. Reporting a case of the giant arterial aneurysm, which remained asymptomatic and undiagnosed for many years, can be a source of better understanding, evaluation, and management plan for arterial aneurysms.

## Case presentation

**Figure 1:**
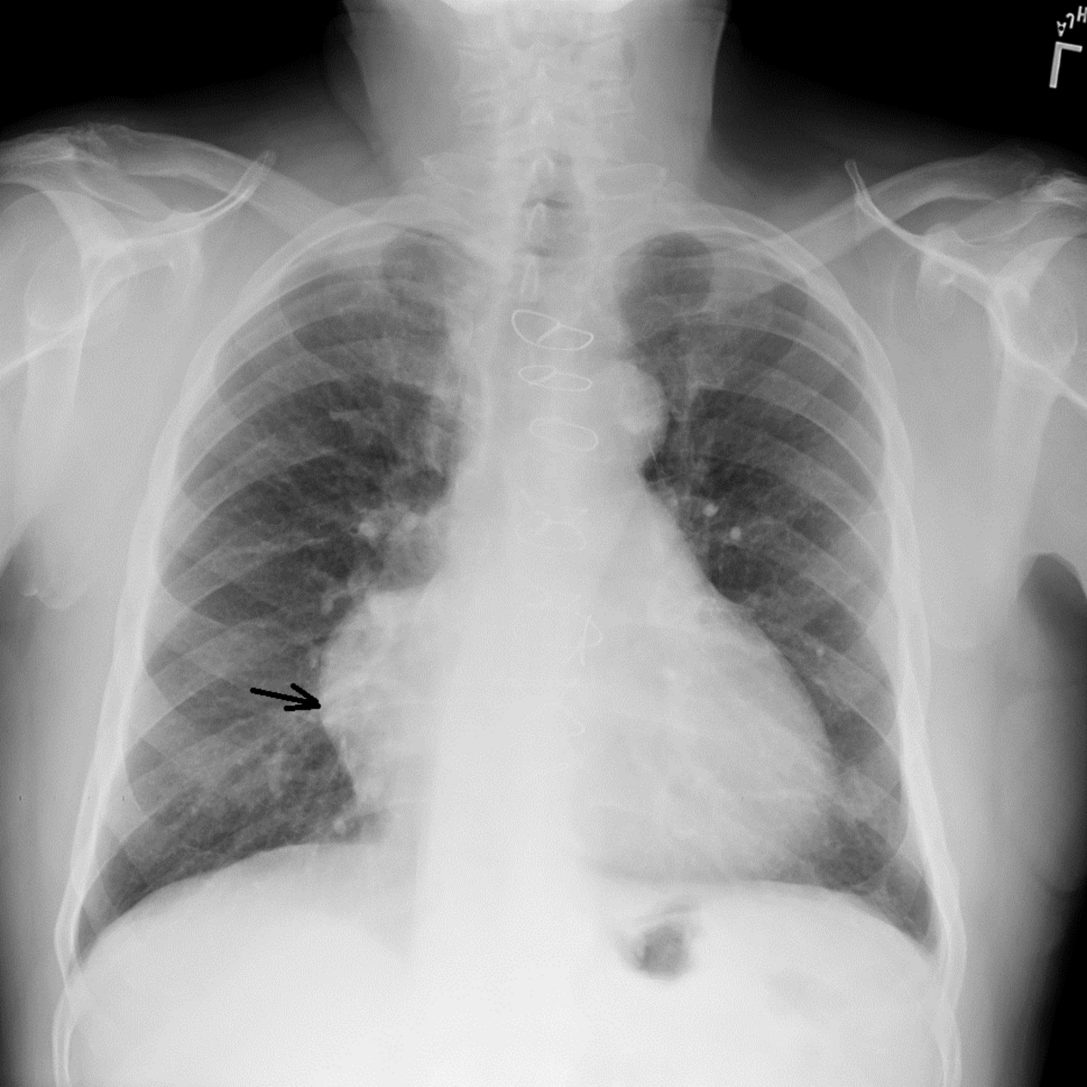
X-ray showing the abnormal contour (black arrow) of the right heart border and evidence of recent pneumonic infiltrates.

An 80-year-old African-American man, with the history of myocardial infarction in November 2000 with two-vessel CABG (coronary artery bypass grafting) performed at that time, now presented to the outpatient radiology suite for a chest X-ray, to follow up on recent pneumonia. An X-ray revealed an abnormal contour of the right heart border (Figure 1).

**Figure 2:**
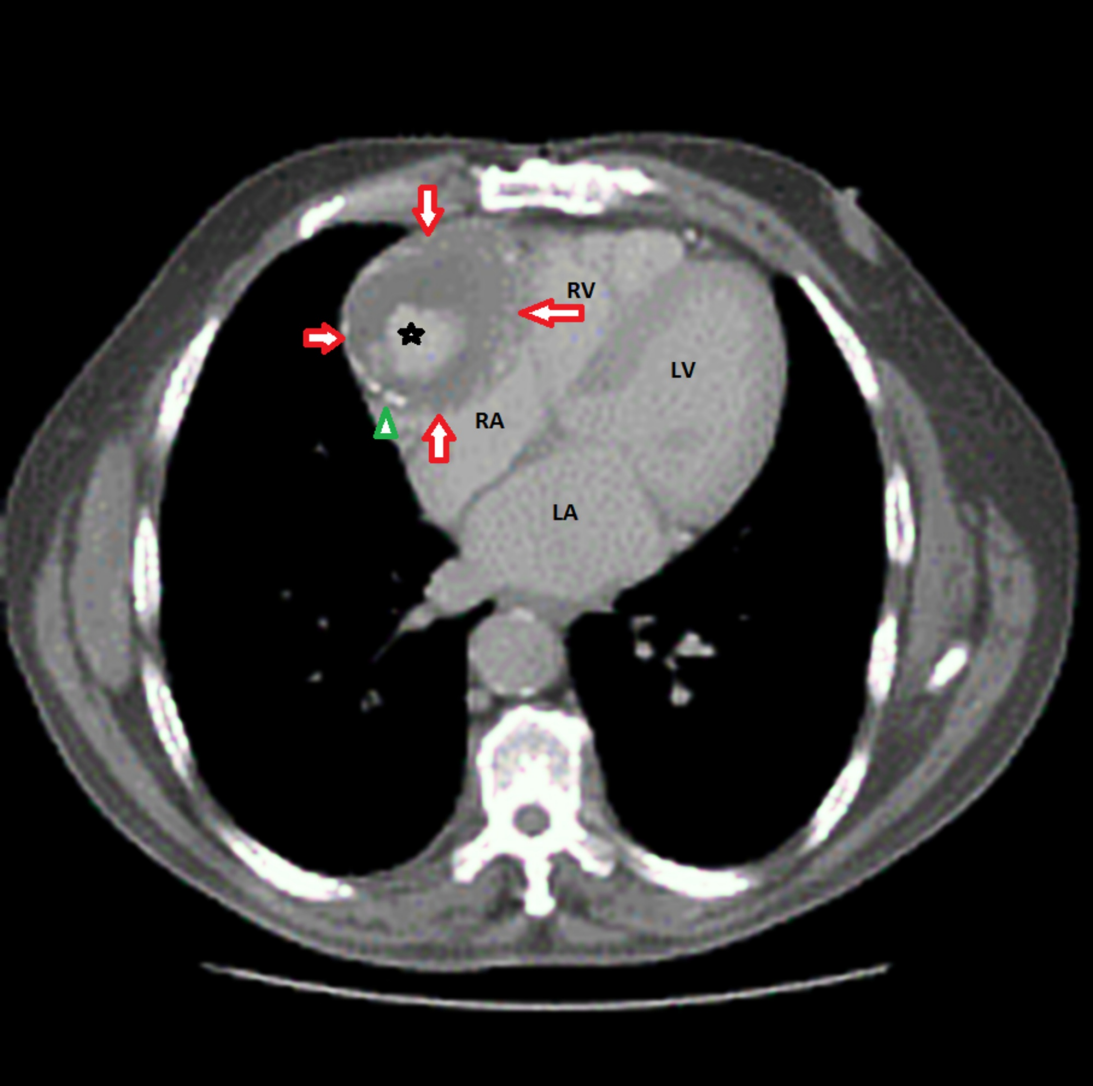
Computed tomography (CAT) scan showing the giant right coronary artery (RCA) aneurysm (red arrows) with partial central thrombosis (asterisk) and intimal calcification (green arrowhead). RV: Right ventricle; RA: Right atrium; LV: Left ventricle; LA: Left atrium.

In order to further investigate the abnormal contouring of the right heart border, a CAT (computed tomography) scan of the chest was performed. Upon checking CAT scan results, the patient was found to have a partially thrombosed aneurysm arising from the native right coronary artery (RCA) measuring approximately 6.9 cm that was also compressing the right atrium (Figure 2).

**Figure 3:**
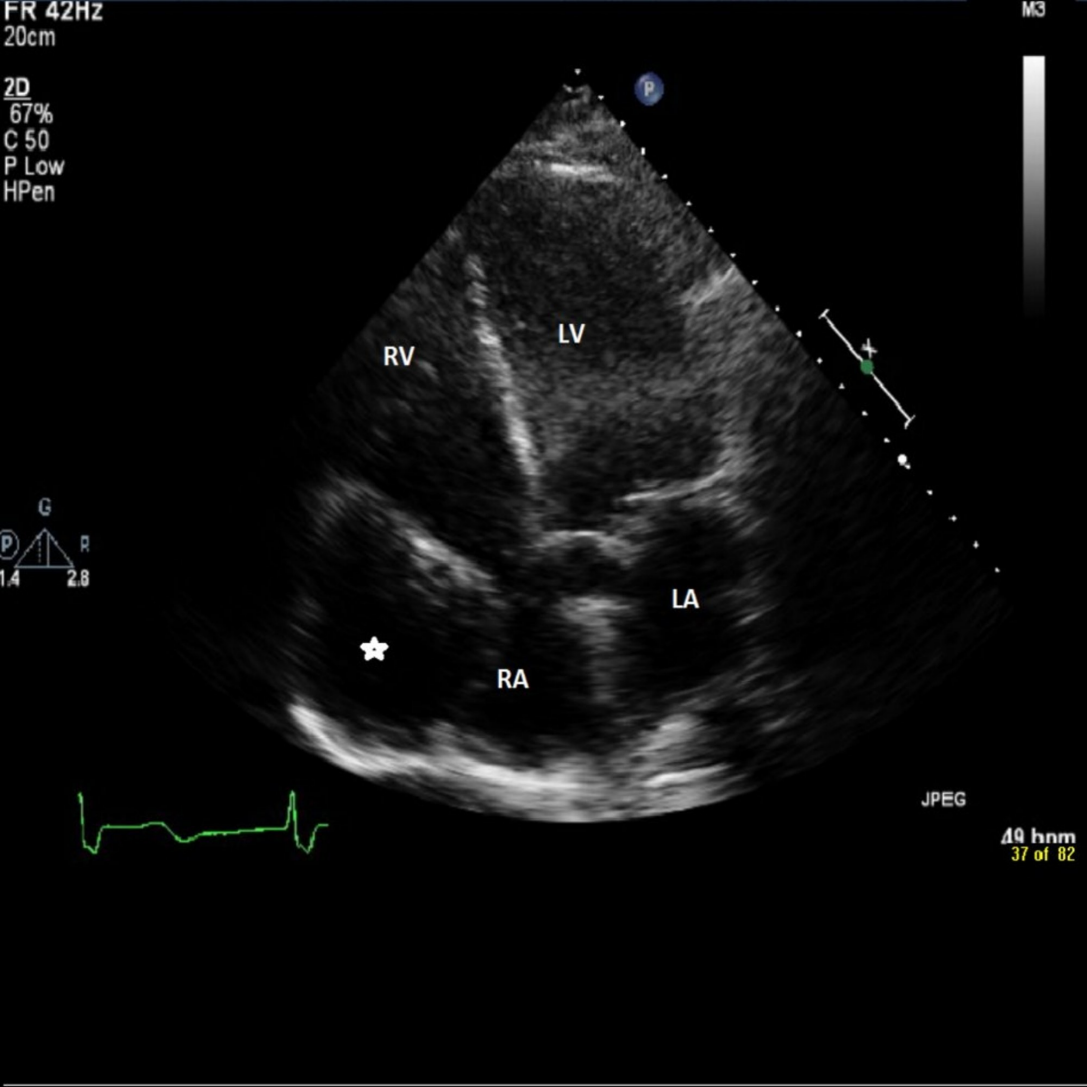
The echocardiogram showing the giant aneurysm (asterisk) arising from the right coronary artery and compressing the right atrium (RA). LA: Left atrium; LV: Left ventricle; RV: Right ventricle.

The patient did not report any symptoms of chest pain, shortness of breath and dizziness. He stated that he was in his usual state of health. He presented with baseline bradycardia with heart rate in the 40s, however, since he was asymptomatic, no further treatment was performed for the bradycardia. Electrocardiogram revealed a bi-fascicular block with Wenckebach. Other labs were normal except for the leucocytosis due to pneumonia (Figure 3).

**Figure 4:**
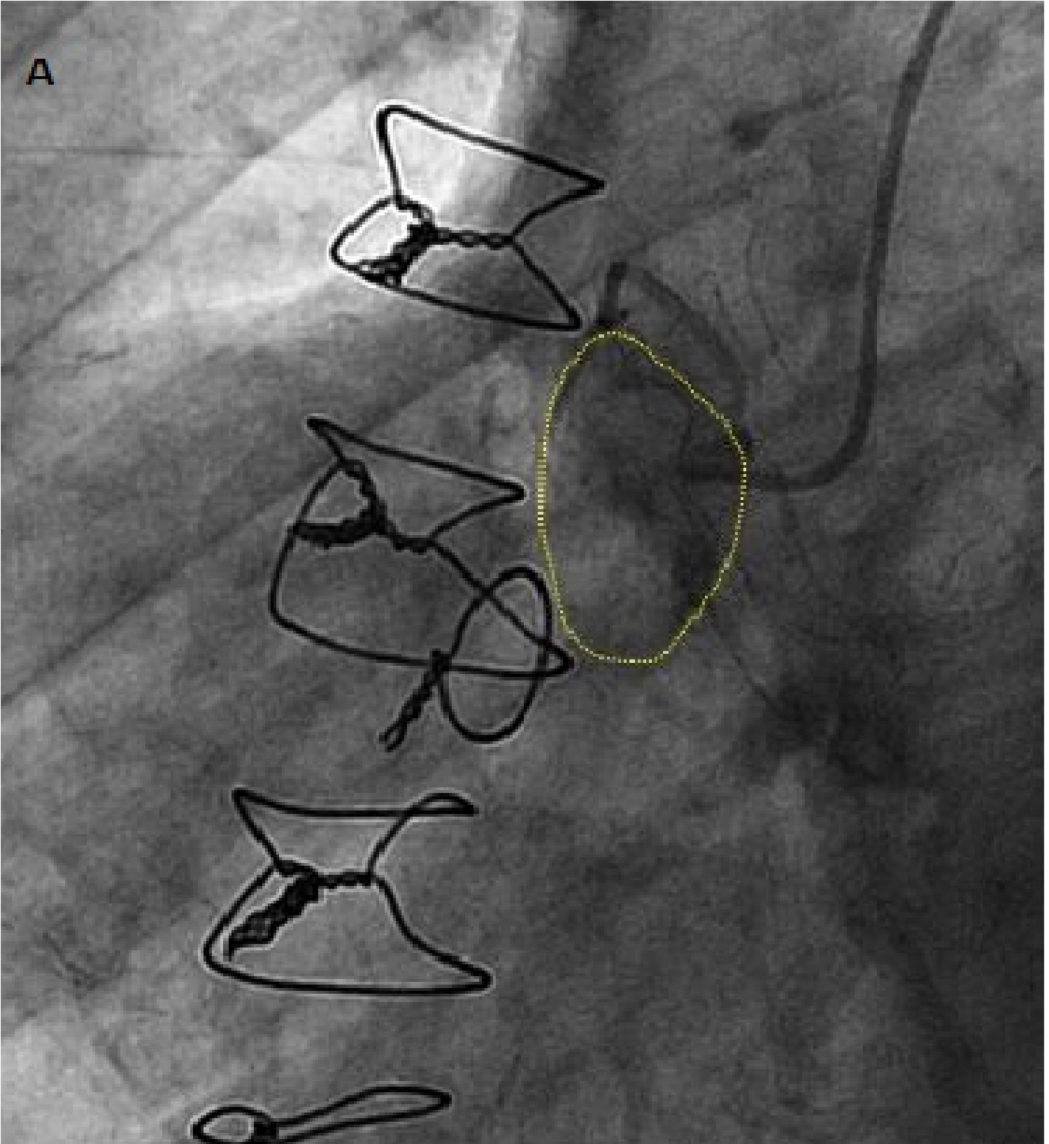
Cardiac catheterization showing native right coronary artery (RCA) aneurysm (yellow dashed line).

Upon initial evaluation and the echocardiography (Figure 3), it was uncertain whether the aneurysm was from the venous graft or the native coronary artery. Thus, it was decided to proceed with the cardiac catheterization to further evaluate the nature of the aneurysm. Catheterization revealed patent LIMA (left internal mammary artery) and RCA grafts along with a native RCA aneurysm (Figure 4).

The case was discussed with cardiothoracic surgeons for interdepartmental consultation and the consensus was that the patient will need a cardiac MRI (magnetic resonance imaging) for the imaging of RCA anatomy prior to intervention. They planned to consult with interventional cardiology for possible covered stent vs. ostial coiling. During the entire eight-day hospital stay, the patient remained completely asymptomatic. He was performing his activities of daily life without any difficulty. Upon discharge, the patient was counseled about the follow-up for outpatient cardiac MRI and to report to the ER if he feels any kind of symptoms including chest pain, dizziness or shortness of breath.

## Discussion

Coronary artery aneurysm is coronary dilatation which exceeds the normal diameter by 1.5 times. When it grows more than 20-150 mm in diameter in adults and more than 8 mm in diameter in children it is called giant arterial aneurysm. The giant coronary aneurysm is a rare condition of coronary artery disease [1]. The incidence of coronary artery aneurysm is 1.5-5% among coronary artery disease [2].

In terms of pathogenesis, it may be caused by atherosclerosis [3-13], Kawasaki disease [14], stent implantation [12], vasculitis [15], autoimmune disease [3], familial hypercholesterolemia [9], congenital in origin [16] or due to blunt chest injury. Regardless of the cause, the pathogenesis is always weakening of the vessel wall which ultimately leads to abnormal dilatation of the vessel.

In terms of symptomatology, it can have various clinical presentations but most commonly it presents with ischemic symptoms leading to angina or myocardial infarction [12]. It can also manifest as superior vena cava syndrome [11], congestive heart failure, compressing right heart [9,10], fistula formation [16], thrombosis, embolism and rupture.

It can be detected by computed tomography, echocardiography, magnetic resonance imaging but coronary angiography remains the gold standard for it which provides information about its size, shape, location and any other associated anomaly. It may be seen as a cardiac tumor, pericardial tumor, and mediastinal mass [4,5,7,8,13].

Its treatment depends upon the symptomatology. Usually, in case of the symptomatic giant arterial aneurysm, surgical treatment is the best option. But in asymptomatic cases, medical management is a better choice (antiplatelet agents, anticoagulation). Prognosis of the coronary arterial aneurysm is controversial, but overall five-year survival is reported in 71% of cases [17,18].

## Conclusions

The reported case highlights that the giant coronary artery aneurysm may not have any signs and symptoms. It should be considered in differentials of any condition mimicking symptoms like those of angina, myocardial infarction, congestive heart failure, etc. and requires proper imaging studies for further evaluation. Management should be planned accordingly.
